# Predictive value of cuproptosis and disulfidptosis-related lncRNA in head and neck squamous cell carcinoma prognosis and treatment

**DOI:** 10.1016/j.heliyon.2024.e37996

**Published:** 2024-09-17

**Authors:** Hongming Liao, Benchao He

**Affiliations:** Department of Otolaryngology Head and neck surgery, Tianmen first people's Hospital, Tianmen, Hubei, 431700, China

**Keywords:** Head and neck squamous cell carcinoma, Immunotherapy, prognosis, Cuproptosis, Disulfidptosis

## Abstract

**Objective:**

Head and neck squamous cell carcinoma (HNSCC) is a highly lethal and prevalent malignant tumor with a poor prognosis due to its high recurrence rate, This study aims to develop a prognostic index for HNSCC patients based on Cuproptosis and Disulfidptosis-related long noncoding RNA.

**Methods:**

Gene expression and clinical data for HNSCC were obtained from The Cancer Genome Atlas (TCGA). Using Lasso regression and multivariate Cox regression, we established a risk scoring model. The predictive ability of the nomogram, based on clinical features and risk scores, was verified using receiver operating characteristics and calibration curves. We compared independent prognostic parameters, risk score distribution, and survival between high-risk and low-risk groups, followed by preliminary validity evaluations of the model.

**Results:**

Our systematic evaluation of prognostic risk provides a new direction for improving the survival prognosis of HNSCC patients in clinical practice, The model effectively categorized patients into high- and low-risk groups with distinct outcomes, identifying numerous gene mutations in these groups, A low-risk score was associated with a better prognosis and higher survival rates.

**Conclusion:**

The risk score prognostic prediction system developed in this study shows potential efficacy in predicting the prognosis of HNSCC patients and has practical applications in clinical settings.

## 1 Introduction

Head and neck squamous cell carcinoma (HNSCC) is a prevalent type of malignancy within the head and neck region. Despite advancements in treatment, HNSCC has a poor prognosis, with high recurrence and metastatic rates contributing to an overall survival rate of approximately 50 % [1,2]. These cancers commonly arise in the oral cavity, oropharynx, nasopharynx, hypopharynx, and larynx. While tobacco and alcohol use are well-established risk factors, the exact etiology of HNSCC remains unclear. Current treatments, which include surgery, radiotherapy, and chemotherapy [3], have shown limited success, highlighting the need for more effective prognostic models to guide clinical management [4].

Cell death processes play a crucial role in various physiological functions, including development, tissue homeostasis, and pathogen defense. Recently, novel forms of regulated cell death such as cuproptosis and disulfidptosis have been identified. Cuproptosis is induced by the buildup of copper, which interacts with lipidated components of the tricarboxylic acid cycle, resulting in cell death [5], Copper ions are involved in various biological functions and are linked to several diseases related to disruptions in copper homeostasis, including inflammation, neurodegenerative disorders, and cancer [[6], [7], [8]]. Disulfidptosis, another form of regulated cell death, involves the accumulation of disulfides, leading to disulfide stress and eventual disulfide poisoning. This process is characterized by the accelerated depletion of nicotinamide adenine dinucleotide phosphate (NADPH) in the cytoplasm due to high expression of SLC7A11 under glucose starvation conditions [9]. Disulfides are relatively stable products that maintain the secondary, tertiary, and quaternary structures of proteins by cross-linking between and within subunits, thereby providing physical and chemical stability to proteins [10]. Therefore, understanding how disulfide accumulation leads to cell death is particularly significant. Despite their potential importance, the roles of cuproptosis and disulfidptosis in HNSCC are not yet well understood.

Zhang [11] developed a prognostic lncRNA atlas associated with cuproptosis to predict responses to immunotherapy, which may offer new potential non-apoptotic therapeutic perspectives for HCC patients. In this study, we utilized data from The Cancer Genome Atlas (TCGA) to identify lncRNAs associated with cuproptosis and disulfidptosis that are differentially expressed in HNSCC. We identified 22 lncRNAs significantly linked to HNSCC prognosis and developed a predictive model based on these findings. Additionally, we examined how this prognostic model correlates with various clinical characteristics, including mutation load. By investigating the molecular mechanisms of cuproptosis and disulfidptosis related lncRNAs in HNSCC progression, we aim to uncover their diagnostic and prognostic values and provide insights into improving clinical outcomes for HNSCC patients.

## 2 Materials and methods

### 2.1 Data acquisition

**Fig. 1:**
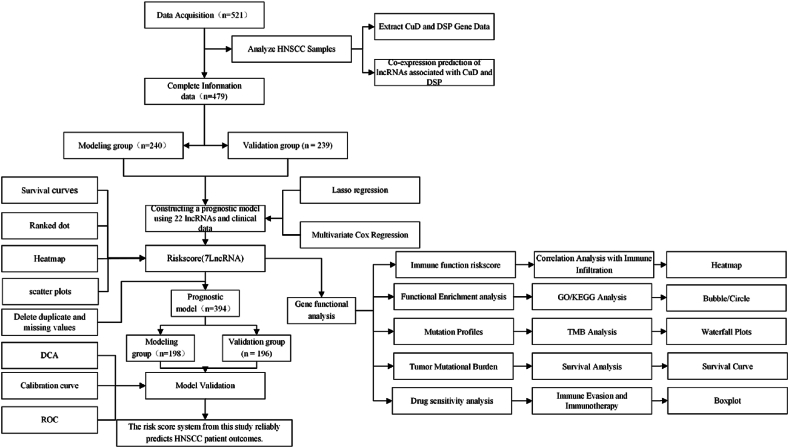
Workflow for Prognostic Prediction and Analysis in HNSCC Using lncRNA and mRNA Expression Data.

This study received approval from the Ethics Committee of The First People's Hospital of Tianmen. We acquired data from The Cancer Genome Atlas (TCGA) (http://tcga-data.nci.nih.gov/), which included lncRNA and miRNA gene expression profiles, as well as clinical information related to head and neck squamous cell carcinoma (HNSCC). A total of 521 HNSCC samples were included in the analysis. From these samples, we extracted gene expression data associated with cuproptosis (CuD) and disulfidptosis (DSP) for subsequent analysis, The detailed methodology is presented in Fig. 1.

### 2.2 Construction prognosis signature

Cuproptosis-related and disulfidptosis-related gene mRNA and lncRNA expression data were extracted and their correlations were analyzed. The samples were randomly divided into two groups: a modeling group (n = 240) and a validation group (n = 239). A risk assessment model was developed using LASSO regression and Cox proportional hazards regression to identify prognostic signatures.

### 2.3 Validation prognosis signature

A multivariate Cox regression model was constructed to evaluate the independent prognostic significance of the parameters. The discrimination ability of the prediction model and scoring system was assessed using the receiver operating characteristic (ROC) curve. Model calibration was evaluated graphically with calibration plots. Decision curve analysis (DCA) was conducted to assess the clinical utility of the decision-making model. Additionally, a nomogram was used to clearly present the prognostic model.

### 2.4 Clinical analysis and construction of the nomogram

We compared risk scores across various clinical features, including age, alcohol, cigarette, gender, T stage, N stage, M stage, and treatment type. Multivariate Cox regression analysis was performed to evaluate the independent prognostic factors. A nomogram was used to clearly present the prognostic model.

### 2.5 Immune function risk score

Each patient's risk score was calculated based on the expression levels of each prognostic lncRNA and its corresponding coefficient. Patients were then classified into low-risk and high-risk groups based on the average riskscore. Additionally, correlation analysis was conducted to assess the relationship between immune cell infiltration in tumor tissues and the risk score system.

### 2.6 Functional enrichment analysis

Gene differential expression analysis was conducted using the limma package in R. Enrichment analysis was performed with the clusterProfiler package, incorporating additional tools such as org. Hs.eg.db, enrichplot, ggplot2, circlize, dplyr, ggpubr, and ComplexHeatmap. This analysis included Gene Ontology (GO) functional annotation—covering biological processes (BP), molecular functions (MF), and cellular components (CC)—as well as KEGG pathway enrichment analysis. The results were illustrated through various plots to effectively convey the findings.

### 2.7 Mutation profiles

The Mutation Annotation Format (MAF) file for HNSCC patients was obtained from the TCGA data portal. Somatic mutations were extracted using the Maftools package in R. Tumor mutation burden (TMB) analysis was performed to compare the mutational burden between high-risk and low-risk groups. Somatic mutation data from TCGA were analyzed using the Maftools R package. Genes with the highest mutation frequency were evaluated in both high-risk and low-risk groups within the TCGA-HNSCC cohort, Waterfall plots were generated to visualize gene mutations in these groups. Additionally, immune escape and immune therapy responses were calculated for the high-risk and low-risk groups using the online tool available at http://tide.dfci.harvard.edu/.

### 2.8 Drug sensitivity analysis

Drug sensitivities for several common medications were evaluated using the Genomics of Drug Sensitivity in Cancer database (https://www.cancerrxgene.org/) through the pRRophetic package [12,13]. The study compared the drug responses between high-risk and low-risk groups, utilizing the pRRophetic package to assess and compare their half-maximal inhibitory concentration (IC50) as a measure of drug sensitivity.

### 2.9 Statistical analysis

Statistical analyses were performed using SPSS Version 25.0 and R software Version 4.2.2. Count data were compared using the Chi-square test or Fisher's exact probability test. LASSO regression was employed to screen for influencing factors, followed by multivariate Cox analysis using the survival package. Kaplan-Meier (KM) survival curves were generated using the R package survminer,A P-value of 0.05 was considered statistically significant.

## 3 Results

### 3.1 Extract target genes

A total of 521 HNSCC samples were analyzed, differentially expressed genes in mRNA and lncRNA datasets were identified using R software version 4.2.2. Cuproptosis-related and disulfidptosis-related genes were obtained from the literature [14,15], The cuproptosis-related genes include DLD, PDHB, ATP7B, ATP7A, DLAT, DLST, SLC31A1, DBT, FDX1, LIPIT1, LIAS, GCSH, and PDHA1; The disulfidptosis-related genes include SLC7A11, FLNA, FLNB, MYH9, TLN1, ACTB, MYL6, MYH10, CAPZB, DSTN, IQGAP1, ACTN4, PDLIM1, CD2AP, and INF2.

### 3.2 Coexpression of mRNA and lncRNA

**Fig. 2:**
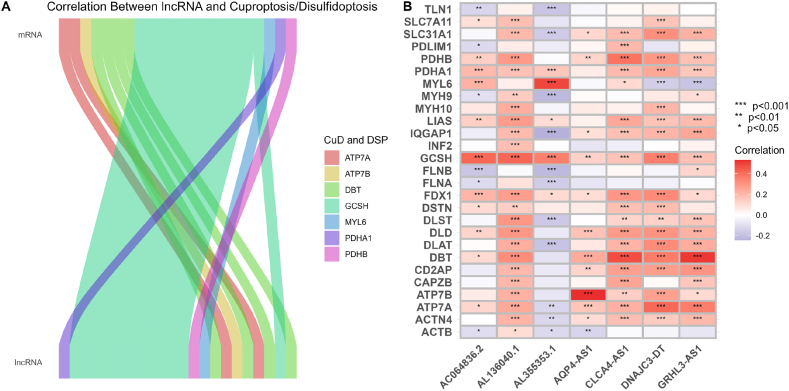
**Correlation analysis between mRNA and lncRNA.** (A) Sankey diagrams of mRNA and lncRNA; (B)Correlations between mRNA and lncRNA.

We extracted and sequenced mRNA, then intersected differential lncRNA targets with mRNA. The final selected mRNAs were PDHA1, GCSH, PDHB, MYL6, DBT, ATP7A, and ATP7B, The final selected lncRNAs included AC005523.1, AC010997.4, AC011773.4, AC025166.1, AC064836.2, AC090517.2, AC093635.1, AC108860.2, AC124067.2, AL136038.4, AL136040.1, AL161756.1, AL353708.1, AL355353.1, AL391840.3, AL442067.3, AQP4-AS1, CLCA4-AS1, DNAJC3-DT, GRHL3-AS1, PRRT3-AS1, and SOX21-AS1. As illustrated in Fig. 2A, the expression levels of mRNA and lncRNA were estimated. A heatmap was generated to depict the correlation between mRNA and lncRNA, with positive correlations shown in red and negative correlations in blue. A p value less than 0.05 is represented by “∗”, a p value under 0.01 by “∗∗”, and one below 0.001 by “∗∗∗”, The heatmap of differentially expressed mRNA and lncRNA is presented in Fig. 2B.

Furthermore, the upper section of the Sankey diagram displays the expression of mRNA genes associated with cuproptosis and disulfidoptosis. The lower section represents lncRNAs that are co-expressed with these mRNAs, with connections indicating significant correlations.

### 3.3 Construction prognosis signature

Patients with HNSCC who had available survival data and lncRNA expression profiles were selected for the study, The samples were randomly divided into two groups: a training group (n = 240) and a validation group (n = 239), The primary purpose of Table 1 is to demonstrate that there are no statistically significant differences between the clinical data of the training and validation groups, thus supporting the validity of using the validation group data to verify the model built from the training group.

**Table 1:** General information comparison between training and validation groups.

Covariates	Type	Total	Validation	Training	P value
Age (years)	<60	214 (44.68 %)	102 (42.68 %)	112 (46.67 %)	0.4318
	≥60	265 (55.32 %)	137 (57.32 %)	128 (53.33 %)	
alcohol	Missing value	8 (1.67 %)	5 (2.09 %)	3 (1.25 %)	0.7153
	no	151 (31.52 %)	77 (32.22 %)	74 (30.83 %)	
	yes	320 (66.81 %)	157 (65.69 %)	163 (67.92 %)	
cigarettes	no	211 (44.05 %)	106 (44.35 %)	105 (43.75 %)	0.9677
	yes	268 (55.95 %)	133 (55.65 %)	135 (56.25 %)	
gender	female	120 (25.05 %)	59 (24.69 %)	61 (25.42 %)	0.937
	male	359 (74.95 %)	180 (75.31 %)	179 (74.58 %)	
T stage	Missing value	4 (0.84 %)	2 (0.84 %)	2 (0.83 %)	0.941
	T1-T2	175 (36.53 %)	89 (37.24 %)	86 (35.83 %)	
	T3-T4	288 (60.13 %)	143 (59.83 %)	145 (60.42 %)	
	unknow	12 (2.51 %)	5 (2.09 %)	7 (2.92 %)	
N stage	N0-N1	299 (62.42 %)	149 (62.34 %)	150 (62.5 %)	0.1935
	N2-N3	153 (31.94 %)	79 (33.05 %)	74 (30.83 %)	
	Missing value	9 (1.88 %)	6 (2.51 %)	3 (1.25 %)	
	unknow	18 (3.76 %)	5 (2.09 %)	13 (5.42 %)	
M stage	M0	449 (94.73 %)	223 (94.49 %)	226 (94.96 %)	0.1357
	M1	6 (1.27 %)	1 (0.42 %)	5 (2.1 %)	
	unknow	19 (4.01 %)	12 (5.08 %)	7 (2.94 %)	
treatment	Pharmaceutical	229 (47.81 %)	112 (46.86 %)	117 (48.75 %)	0.7473
	Radiation	250 (52.19 %)	127 (53.14 %)	123 (51.25 %)	

Legend: Comparison of general information between the training group (n = 240) and the validation group (n = 239), showing no statistically significant differences in key demographic and clinical characteristics. This ensures that the validation group can effectively be used to validate the model built from the training group.

∗ Missing value indicates that the group information is missing; Unknow indicates that the information situation of this group is unclear.

**Fig. 3:**
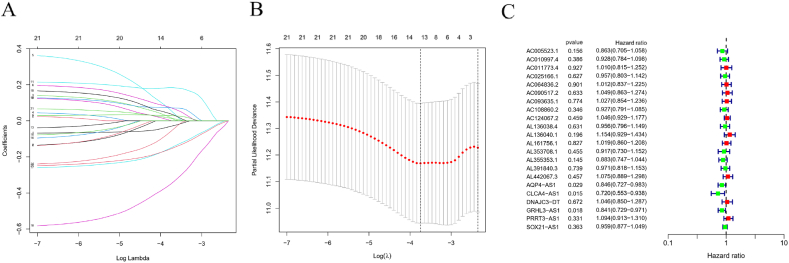
**Construction of the prognostic model.** (A) LASSO coefficient diagram for prognostic genes; (B) Cross‐validation error curve for modeling gene identification(C) Related LncRNA Forest Map.

To evaluate prognostic significance, we first identified 22 lncRNAs associated with mRNA and combined them with survival data. We conducted 10-fold cross-validation using the COX-LASSO regression model, followed by multivariate COX regression analysis, as illustrated in Fig. 3(A、B). From this analysis, COX-LASSO regression selected the smallest λ independent variable (14), which was then used for multivariate COX regression analysis, ultimately identifying 7 important lncRNAs. The risk score was calculated by adding the products of the regression coefficients and gene expression levels. The regression coefficients and related lncRNAs are shown in Table 2. The median risk score was used to divide the patients into high-risk and low-risk groups for further analysis. Additionally, a forest plot displaying the univariate COX regression results for the 22 lncRNAs is provided in Fig. 3C.

**Table 2:** LncRNAs and their coefficients were identified after multivariate COX regression.

lncRNA	Coefficients
AC064836.2	0.335748007
AL136040.1	0.285865693
AL355353.1	−0.229229159
AQP4-AS1	−0.226420495
CLCA4-AS1	−0.503683916
DNAJC3-DT	0.20899865
GRHL3-AS1	−0.245596924

### 3.4 Survival analysis heatmap

**Fig. 4:**
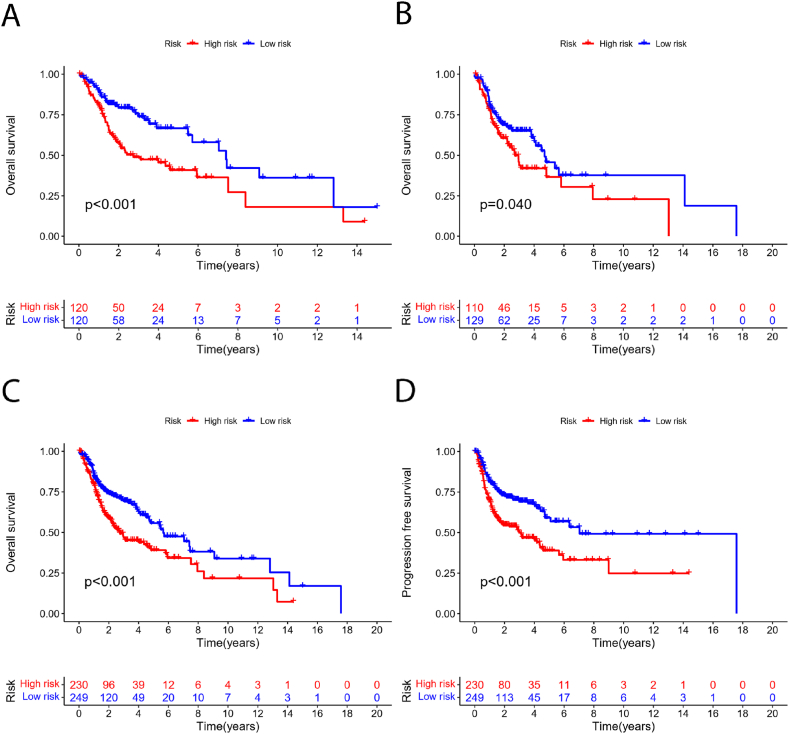
**Cuproptosis and Disulfidptosis-related prognostic signature.** (A) Ranked dot, scatter plots and heat map of the model gene expressions in the all patients; (B) Ranked dot, scatter plots and heat map of the model gene expressions in the modeling group; (C) Ranked dot, scatter plots and heat map of the model gene expressions in the validation group.

Cancer patients were divided into low-risk and high-risk groups based on their risk scores, with red and blue colors representing high and low risk scores, respectively. Fig. 4 shows the progression-free and overall survival curves for both the low-risk and high-risk groups. These curves reveal that patients classified as low-risk have significantly better progression-free and overall survival rates compared to those classified as high-risk. Fig. 4 illustrates the survival curves for the training group, validation group, and the overall patient population. These curves indicate that the risk stratification model accurately differentiates between various risk levels within each group. The low-risk group consistently exhibits superior survival outcomes compared to the high-risk group across all cohorts, validating the effectiveness and reliability of the model.

**Fig. 5:**
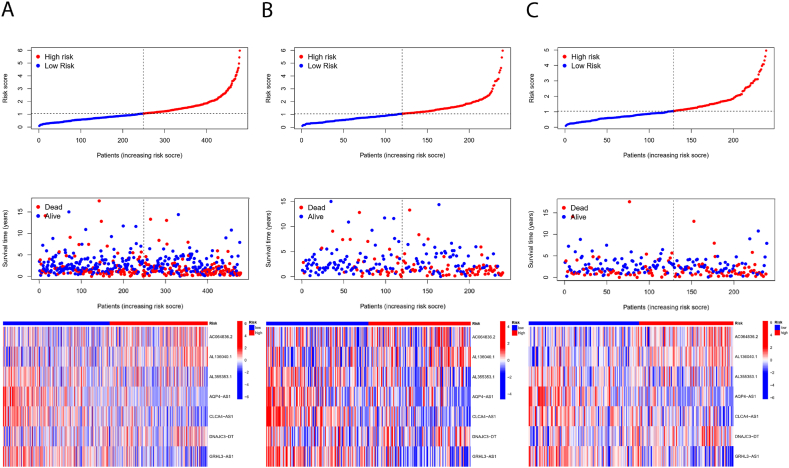
**Cuproptosis and Disulfidptosis-related prognostic signature.** (A) Ranked dot, scatter plots and heat map of the model gene expressions in the all patients; (B) Ranked dot, scatter plots and heat map of the model gene expressions in the modeling group; (C) Ranked dot, scatter plots and heat map of the model gene expressions in the validation group.

Fig. 5 illustrates the prognostic features related to cuproptosis and disulfidptosis. Fig. 5A presents a dot plot, scatter plot, and heatmap of the model gene expression for all patients. Fig. 5B shows the ranked dot plot, scatter plot, and heatmap of model gene expression in the training group. Fig. 5C provides the dot plot, scatter plot, and heatmap of model gene expression in the validation group.

In summary, these findings underscore the prognostic significance of the identified lncRNAs and their capacity to effectively categorize patients into risk groups that align with their survival prospects.

### 3.5 Analysis of independent prognoses

Using LASSO regression and multivariate Cox regression analyses, we retained seven significant lncRNAs: AC064836.2, AL136040.1, AL355353.1, AQP4-AS1, CLCA4-AS1, DNAJC3-DT, and GRHL3-AS1. Their corresponding coefficients are 0.336, 0.286, −0.229, −0.226, −0.504, 0.209, and −0.246, respectively, Samples without clinical data were excluded from the analysis, and patients missing prognostic time and status were removed. Consequently, 394 out of 479 individuals were selected for univariate and multivariate Cox regression analyses. The results indicated that cigarette, M stage, gender, and risk score were independent prognostic factors for overall survival (OS), as detailed in Table 3.

**Table 3:** HNSCC using univariate and multivariate cox regression analysis.

Variable	Univariate ananlysis		Multivariate analysis	
	HR（95%CI）	P value	HR（95%CI）	P value
Alcohol	1.221（0.709–2.102）	0.471		
Cigarettes	1.752（1.048–2.928）	0.033	1.843（1.087–3.126）	0.023
Age	1.626（0.993–2.663）	0.053		
gender	0.484（0.298–0.787）	0.003	0.481（0.294–0.788）	0.004
T stage	1.179（0.723–1.921）	0.51		
N stage	1.174（0.749–1.84）	0.485		
M stage	3.532（0.86–14.514）	0.08	4.114（0.976–17.344）	0.054
Treatment	1.352（0.826–2.214）	0.231		
RiskScore	1.848（1.504–2.27）	＜0.05	1.856（1.501–2.295）	＜0.05

### 3.6 Validation prognosis signature

**Fig. 6:**
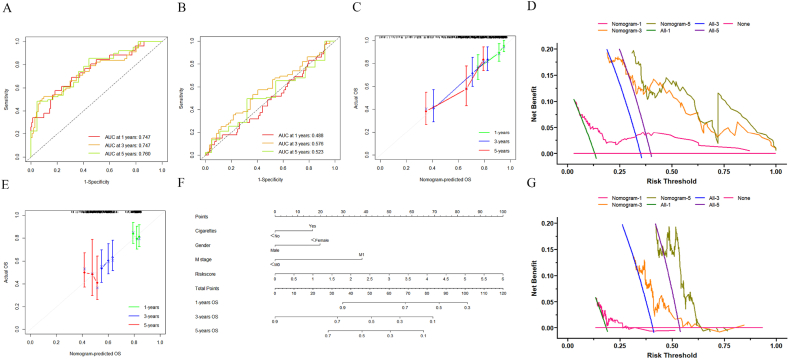
**Establishment and verification of the nomogram**.(A) Roc curve (Modeling group); (B)Roc curve (Validation group); (C)Calibration curve (Modeling group); (D)DCA curves (Modeling group); (E)Calibration curve (Validation group); (F)Nomogram; (G)DCA curves (Validation group).

A multivariate Cox regression model was developed to assess the independent prognostic significance of various parameters. The nomogram, which includes cigarettes, gender, M stage, and riskscore, is illustrated in Fig. 6. This nomogram effectively represents the prognostic model. ROC curves for 1-year, 3-year, and 5-year survival rates in both the modeling and validation groups are shown in Fig. 6A and B. The concordance index (C-index) for the modeling group is 0.70, while the validation group shows a C-index of 0.53, To assess the accuracy of the nomogram, calibration plots for 1-year, 3-year, and 5-year intervals were generated. Both the modeling and validation groups exhibited strong agreement between predicted and actual survival probabilities (Fig. 6C and E). Additionally, the clinical utility of the recalibrated model was evaluated through decision curve analysis (DCA), with the DCA curves confirming the clinical applicability of the nomogram (Fig. 6D and G).

### 3.7 Model validation for clinical grouping

**Fig. 7:**
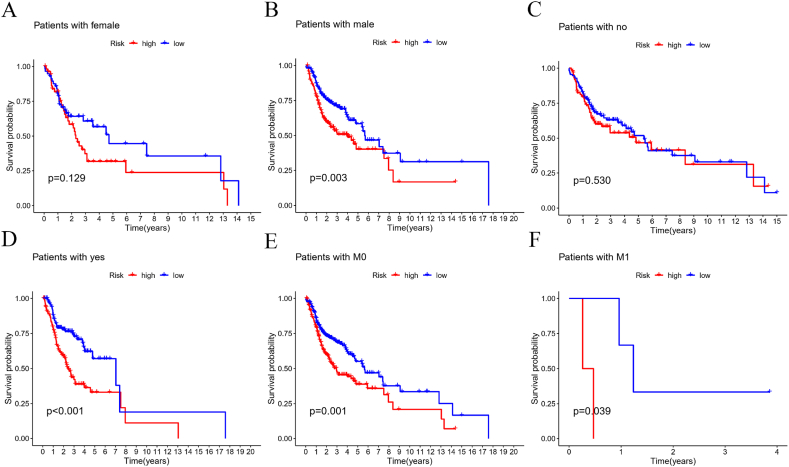
**Patient survival period analysis.** (A,B) Survival probability at different gender; (C,D) Survival probability of different cigarette; (E,F) Survival probability of different M stages.

**Fig. 8:**
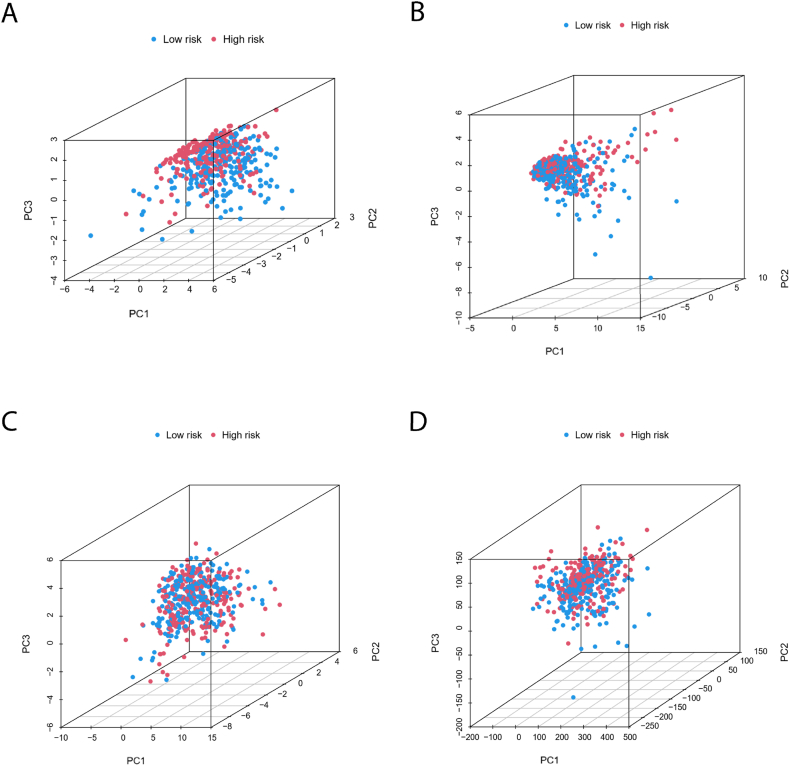
**Principal component analysis.** (A) Model LncRNA; (B) cuproptosis and disulfidptosis related LncRNA; (C) Cuproptosis and disulfidptosis related gene; (D)All genes.

To assess the predictive capability of our model for various clinical variables, we performed validation as depicted in Fig. 7（A – F）, We then examined the influence of clinical factors such as M-stage, cigarettes, and gender on patient prognosis. Our analysis revealed that male gender, M-stage, and cigarettes all have significant statistical associations. Additionally, principal component analysis (PCA) was conducted to determine if the lncRNAs used in our model could effectively differentiate between high-risk and low-risk patients. The results are illustrated in the following figures: Fig. 8A highlights the model lncRNAs, clearly distinguishing between high-risk and low-risk populations; Fig. 8B shows lncRNAs associated with copper and disulfide deposition; Fig. 8C displays genes related to copper and disulfide deposition; and Fig. 8D lists all analyzed genes. Fig. 8A effectively demonstrates how the model lncRNAs differentiate between high-risk and low-risk patients.

### 3.8 Enrichment analysis and immune functional analysis

**Fig. 9:**
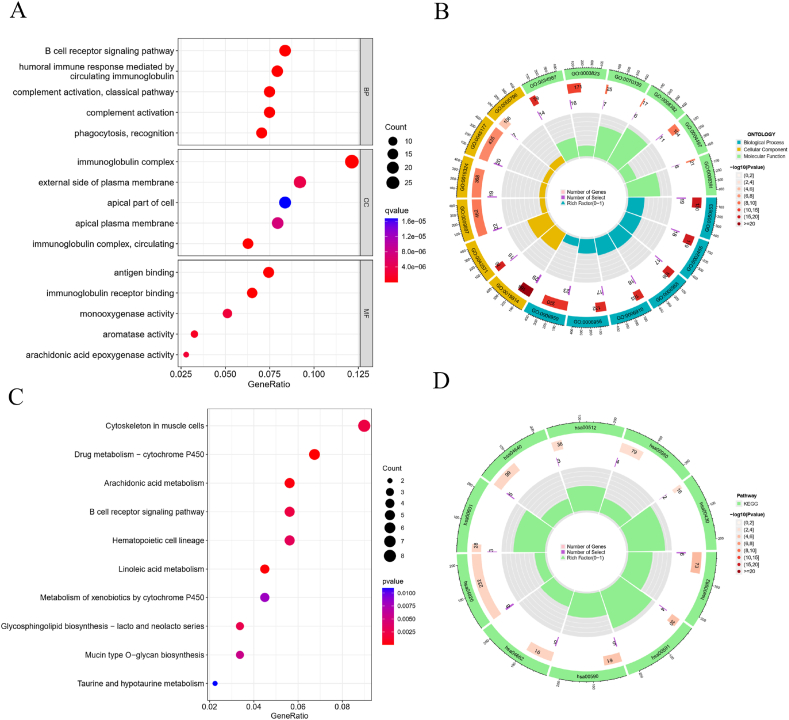
**Immune process regulation in HNSCC.** (A,B) Biological processes (BP), Cellular components (CC) and Molecular functions (MF); (C,D) Kyoto Encyclopedia of Genes and Genomes (KEGG) pathway.

We conducted a differential analysis to identify risk genes distinguishing high-risk from low-risk populations. This analysis included enrichment of Gene Ontology (GO) categories—biological processes (BP), molecular functions (MF), and cellular components (CC)—covering a total of 70 terms. The primary enriched processes were related to the human immune response, including circulating immunoglobulin, humoral immune response mediated by circulating immunoglobulin, complement activation via the classical pathway, immunoglobulin complex, external side of the plasma membrane, immunoglobulin receptor binding, antigen binding, and aromatase activity. To visualize these findings, we utilized the ClusterProfiler, Enrichplot, and ggplot2 packages to create circle and bubble charts, highlighting the top 5 entries for GO enrichment in BP, CC, and MF. Additionally, KEGG pathway enrichment analysis was performed, focusing on pathways such as Drug Metabolism-Cytochrome P450, Linoleic Acid Metabolism, Arachidonic Acid Metabolism, B Cell Receptor Signaling Pathway, and Cytoskeleton in Muscle Cells. Using R version and the ClusterProfiler package, we generated circle and bubble plots to illustrate these pathways, as shown in Fig. 9A – D.

### 3.9 Tumor mutational burden and drug sensitivity analyses

**Fig. 10:**
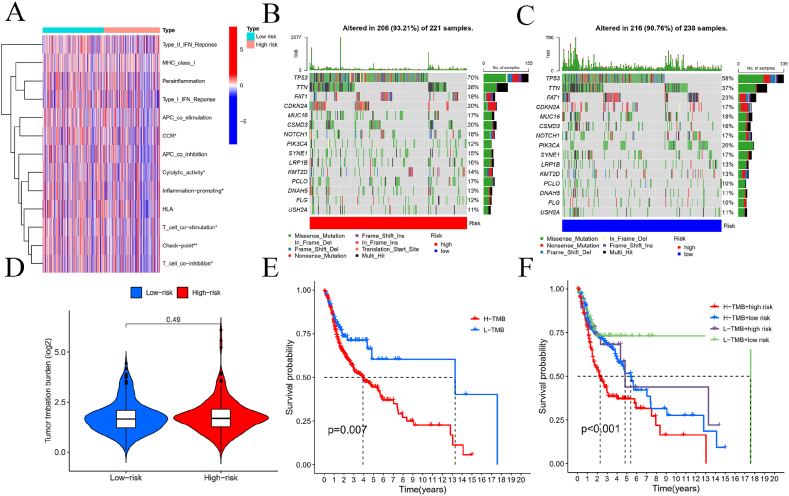
**Immune function and TMB between the high‐risk and low‐risk groups.**(A) Heatmap showed the immune function; (B,C) Differences in mutations between the high‐risk and low‐risk groups; (D) Response rate between high- and low-risk group by TIDE algorithm in TCGA-HNSCC cohort; (E,F) Survival probability analysis combining TMB and risk score.

**Fig. 11:**
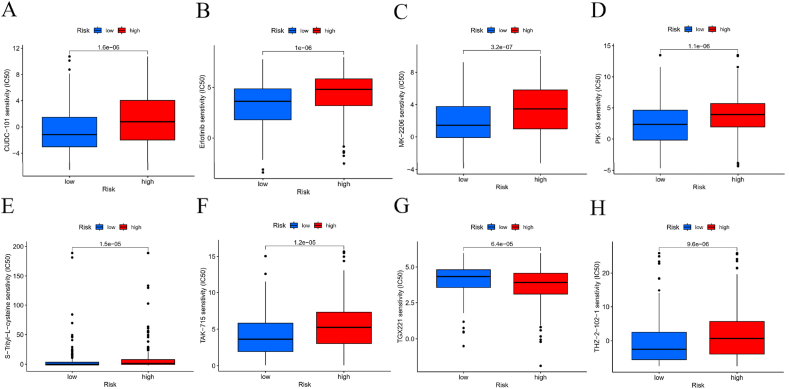
**Drug sensitivity between high- and low-risk.** (A–H) Calculate the half-maximal inhibitory concentration of FDA-approved drugs in the risk model, including, CUDC-100(A), Erlotinib(B),MK-2260(C), PIK-93(D),S-Trityl-L-cysteine(E), TAK-715(F), TGX221(G), and THZ-2-102-1(H).

Fig. 10A illustrates the relationship between various immune cells and high-risk versus low-risk groups. The TIDE score was utilized to evaluate tumor immune evasion. Our study found no significant correlation between risk levels and immune evasion, as depicted in Fig. 10D.Based on our predictions, we analyzed gene mutation frequencies in low-risk and high-risk populations. Fig. 10B presents a waterfall plot of gene mutation frequencies for the high-risk group, while Fig. 10C shows the corresponding plot for the low-risk group. Generally, the high-risk group exhibited a higher frequency of gene mutations compared to the low-risk group. Tumor mutation data were used to categorize patients into high and low mutation burden groups. Survival analysis demonstrated that the low mutation burden group had a better prognosis compared to the high mutation burden group, as shown in Fig. 10E.Furthermore, a combined survival analysis of tumor mutations and patient risk further highlighted significant differences, as illustrated in Fig. 10F.Additionally, to enhance the clinical applicability of the risk score, we utilized the pRRophetic package to assess the sensitivity of different patient groups to HNSCC treatments. Fig. 11A – H displays the values for eight relevant drugs in the high-risk population.

## 4 Discussion

Head and neck squamous cell carcinoma (HNSCC) is a prevalent malignancy characterized by significant heterogeneity and a high mortality rate [[16], [17], [18]]. Due to the often subtle and nonspecific symptoms, HNSCC is frequently diagnosed at advanced stages, complicating early intervention. The progression of cancer cells, including their metabolic alterations and genetic reprogramming during malignant transformation, is closely linked to interactions with the tumor microenvironment [19]. Understanding the roles of cuproptosis and disulfidptosis-related long noncoding RNAs in HNSCC is crucial, as these mechanisms are involved in key processes such as metabolism, proliferation, migration, and apoptosis of tumor cells. Specifically, cuproptosis, which is triggered by copper accumulation, leads to cellular stress and death, while disulfidptosis, caused by elevated disulfide levels, results in oxidative stress and subsequent cell damage. These cell death mechanisms could influence tumor behavior and progression, potentially serving as prognostic biomarkers for HNSCC. However, the relationship between these long noncoding RNAs and HNSCC remains insufficiently explored.

Through the analysis of the TCGA dataset, we identified lncRNAs associated with cuproptosis and disulfidptosis in HNSCC. Our subsequent aim was to develop a prognostic model for HNSCC based on these lncRNAs using the LASSO method. This model is designed to predict the one-year, three-year, and five-year survival rates of HNSCC patients. The ROC curve, nomogram, and calibration analysis of the model have demonstrated substantial clinical predictive accuracy. To enhance the model's practical relevance, we recommend integrating it into clinical workflows by incorporating it into electronic health records (EHRs) to assist in real-time survival predictions and treatment planning. Clinicians could use the nomogram to assess individual patient risk profiles and make informed decisions regarding personalized treatment approaches, However, to address potential overfitting and validate the model's robustness, we propose several future experimental validations. These include: (1) conducting external validation studies with independent datasets to confirm the model's generalizability, (2) performing prospective cohort studies to test its predictive accuracy in real-world clinical settings, and (3) assessing the model's performance across diverse patient demographics and treatment modalities. In addition, the model's integration with other clinical variables such as risk score, cigarettes, gender, and M stage, which were identified as independent prognostic factors with high HR values, provides a comprehensive tool for survival prediction and patient management.

The tumor mutational burden (TMB) is strongly associated with tumor immunity, with a high mutational load generally indicating poorer outcomes in HNSCC patients. Increased mutations lead to higher neoantigen expression, which improves the immune system's ability to detect and attack cancer cells [20]. High-risk patients show markedly different prognoses compared to those at low risk. Tumors with numerous copy number alterations, signifying chromosomal instability, often have mutations in DNA repair genes, worsening the prognosis. Tumors with a high mutational burden are more likely to be malignant [21]. Our meta-analysis found that TP53 mutations significantly affect HNSCC survival, though some limitations exist [22]. These results are consistent with earlier studies. TMB and immune checkpoint molecules are reliable for assessing immunotherapy effectiveness. The low-risk group had the highest immune cell counts, indicating robust immune activation, while high-risk tumors showed increased immune infiltration, offering new insights into tumor-immune interactions. Our analysis also included Gene Ontology (GO) enrichment, revealing key processes related to the immune response, such as circulating immunoglobulin, humoral immune response, complement activation via the classical pathway, immunoglobulin complex, and antigen binding. KEGG pathway enrichment highlighted significant pathways including Drug Metabolism-Cytochrome P450, Linoleic Acid Metabolism, Arachidonic Acid Metabolism, B Cell Receptor Signaling, and Cytoskeleton in Muscle Cells.

Cells can undergo either accidental cell death (ACD) or regulated cell death (RCD) regardless of growth conditions [23]. RCD includes processes such as apoptosis, necroptosis, autophagy, ferroptosis, pyroptosis, Cuproptosis, and Disulfidptosis [24]. This study focuses on Cuproptosis and Disulfidptosis, where tumor cell death is triggered by high levels of disulfides and copper. Long intergenic non-coding RNAs (lncRNAs), which are RNA molecules longer than 200 nucleotides and do not code for proteins, are involved in these processes and contribute to tumor cell apoptosis [[25], [26], [27]]. Notably, Disulfidptosis was identified as a new form of cell death in 2023 [28]. Using TCGA data, we discovered a novel metabolic gene signature that predicts HNSCC prognosis. We observed significant differences in immune infiltration and immune checkpoint genes between high-risk and low-risk HNSCC patients. Inflammation that promotes tumors is a known cancer characteristic [29]. The study identified that lncRNAs linked to Cuproptosis and Disulfidptosis act as negative prognostic biomarkers for HNSCC. Furthermore, these lncRNAs are associated with various other cancers, highlighting their broader relevance in cancer prognosis and potential utility across different malignancies [30,31]. These biomarkers have potential for predicting immunotherapy responses and assisting in patient stratification for HNSCC treatment. Our results suggest that personalized cancer treatment strategies could significantly benefit HNSCC patients, marking a promising direction in clinical cancer management.

Despite its contributions, the study has limitations. To fully validate the molecular mechanisms of long noncoding RNAs related to Cuproptosis and Disulfidptosis in the prognostic model, further experimental research is needed. Our findings were based on bioinformatics analyses, which require confirmation through additional experimental work. There is also a risk of overfitting because the external validation data came from the same cohort, highlighting the need for more robust validation to improve the reliability and accuracy of the results.

## 5 Conclusions

In summary, we developed a predictive risk model for HNSCC patients that provides significant prognostic insights into patient outcomes. This model not only predicts survival rates but also evaluates patients' sensitivity and responsiveness to immunotherapy. Consequently, it serves as a valuable tool for guiding clinical practice and tailoring personalized treatments for HNSCC.

## Ethical statement

This study was approved by the Ethics Committee of The First People's Hospital of Tianmen, Ethics Approval Number:20230139, Full Name of Ethics Committee: Ethics Committee of Tianmen First People's Hospital.

## Data and code availability statement

The authors do not have permission to share data.

## CRediT authorship contribution statement

**Hongming Liao:** Writing – original draft. **Benchao He:** Writing – review & editing.

## Declaration of competing interest

The authors declare the following financial interests/personal relationships which may be considered as potential competing interests: Benchao He reports financial support was provided by Tianmen First People's Hospital. No conflict of interest If there are other authors, they declare that they have no known competing financial interests or personal relationships that could have appeared to influence the work reported in this paper.
